# Durability Tests of a Fiber Optic Corrosion Sensor

**DOI:** 10.3390/s120303656

**Published:** 2012-03-16

**Authors:** Kai Tai Wan, Christopher K.Y. Leung

**Affiliations:** 1 Department of Civil Engineering, Chu Hai College of Higher Education, Riviera Garden, Tsuen Wan, Hong Kong, China; 2 Department of Civil and Environmental Engineering, Hong Kong University of Science and Technology, Clearwater Bay, Hong Kong, China; E-Mail: ckleung@ust.hk

**Keywords:** fiber optic, corrosion sensor, sputtering, OTDR, optical time domain reflectometer, chemical degradation, freeze-thaw cycling

## Abstract

Steel corrosion is a major cause of degradation in reinforced concrete structures, and there is a need to develop cost-effective methods to detect the initiation of corrosion in such structures. This paper presents a low cost, easy to use fiber optic corrosion sensor for practical application. Thin iron film is deposited on the end surface of a cleaved optical fiber by sputtering. When light is sent into the fiber, most of it is reflected by the coating. If the surrounding environment is corrosive, the film is corroded and the intensity of the reflected signal drops significantly. In previous work, the sensing principle was verified by various experiments in laboratory and a packaging method was introduced. In this paper, the method of multiplexing several sensors by optical time domain reflectometer (OTDR) and optical splitter is introduced, together with the interpretation of OTDR results. The practical applicability of the proposed sensors is demonstrated in a three-year field trial with the sensors installed in an aggressive marine environment. The durability of the sensor against chemical degradation and physical degradation is also verified by accelerated life test and freeze-thaw cycling test, respectively.

## 1. Introduction

Steel corrosion is the major culprit for the degradation of reinforced concrete structures. The steel reinforcement is passivated and the corrosion rate is negligible in the high alkaline environment of concrete. However, it is depassivated and the corrosion rate becomes significant when chloride ions or carbon dioxide penetrate to the steel reinforcement through the concrete cover. Therefore, in conventional approach to monitor the steel corrosion, the depth of chloride penetration or carbonation is assessed by the coring of samples from the concrete cover for chemical analysis. A detailed description can be found in Broomfield [1]. Several nondestructive electrochemical measurement techniques were described in [2]. Some of them are commonly applied in practice. However, all the electrochemical approaches are labor-intensive and inapplicable in an environment with significant electromagnetic interference (e.g., from power cable nearby) and most of the methods are only effective after steel corrosion has started.

For prognostic monitoring, Schiessl *et al*. [3] and Raupach [4] in Germany developed a corrosion cell with the shape of a “ladder”. By measuring the corrosion current between a reference and steel rods at different positions in the concrete cover, corrosion penetration can be monitored. However, the corrosion cell is expensive and cannot be retrofitted into existing structures. With small size and immunity to electromagnetic interference, fiber optic sensors are alternatives to the electrochemical approach. Various fiber optic sensors have been developed for monitoring different crucial factors affecting steel corrosion, such as humidity [5], pH [5,6] and chloride content [7,8]. However, the humidity measurements do not directly related to corrosion activity and the pH sensors cannot detect chloride-induced corrosion. Also, the critical chloride content for corrosion initiation is dependent on pH values and the relationship is not well-defined [9].

Leung *et al*. [10] demonstrated the feasibility of a fiber optic corrosion sensor for monitoring the corrosiveness of the concrete environment through various laboratory experiments. For practical application of the sensor to reinforced concrete structures, the durability of the sensor should be verified. In this paper, the sensing principle will be briefly reviewed first, followed by the measurement technique for multiplexed sensors. Three verifications of the sensor durability will be presented. The first is a field trial in which the sensors were installed in an existing reinforced concrete footbridge exposed to an aggressive marine environment. The second is the accelerated life test to verify the durability of the sensor against chemical degradation in a concrete environment. The last one is to test the embedded sensors against physical degradation under freeze-thaw cycling.

## 2. Sensing Principle and Measurement Technique

**Figure 1.**
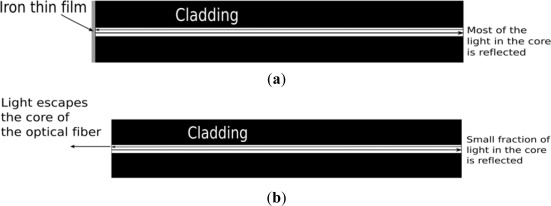
Illustration of the sensing principle. (**a**) The light inside the core of the optical fiber is reflected by the iron thin film; (**b**) The light escape of optical fiber after the iron thin film is depleted.

The proposed sensor does not detect the steel corrosion rate directly. Instead, it monitors the corrosiveness of the surrounding environment. The sensing principle is illustrated in Figure 1. A pure thin iron film, which is about 200 nm thick, is deposited onto the cleaved end of a bare optical fiber by sputtering. The thin iron film reflects the light as a mirror (Figure 1(a)). When the surrounding environment of the sensor is corrosive, the film is depleted and most of the light escapes the optical fiber and the intensity of the reflected light drops significantly.

The proposed sensor possesses a number of advantages compared to other corrosion sensors. The interpretation of the results is easy and direct because of the very simple sensing principle. It can measure any corrosive agents in the surrounding environment, including chloride ion and carbon dioxide. Mass production of the sensors at low cost is feasible because single mode telecommunication optical fiber can be employed and a large number of optical fibers can be sputtered at a time. Moreover, thanks to the small size of the sensor, it can be retrofitted to existing structures.

**Figure 2.**
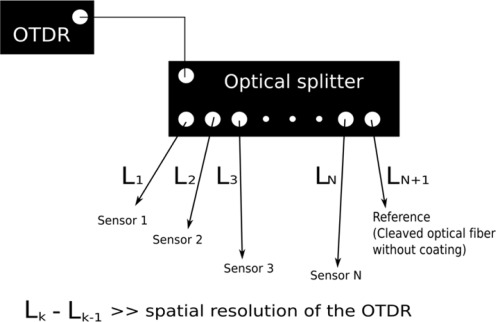
Schematic diagram of using OTDR and optical splitter to measure the reflectivity of several sensors in single OTDR reading.

**Figure 3.**
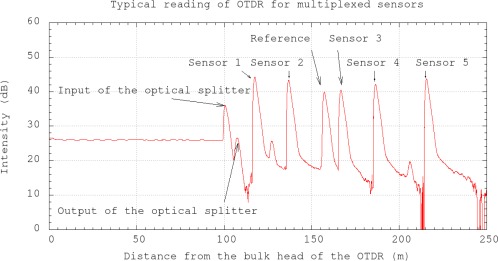
Typical OTDR reading of multiplexed sensors.

Several sensors can be multiplexed with the use of an optical splitter (Figure 2) and the reflectivity at the end of each sensor can be measured with optical time domain reflectometer (OTDR). The principle of the OTDR is that it sends a short laser pulse to the optical fiber and measures the intensity of the reflected light and the corresponding arrival time. When the pulse reaches the fiber end, light is reflected back. By using the optical splitter, the laser pulse from the OTDR travels to different sensors. If the differential distances between each sensor and the optical splitter are all significantly longer than the spatial resolution of the OTDR, the reflectivity of all sensors can be obtained from a single OTDR reading. To eliminate the fluctuation of light source and the insertion losses, a cleaved optical fiber without coating is connected to the optical splitter as a reference. A typical OTDR reading of the multiplexed sensors is shown in Figure 3. The variations of the light source, insertion losses at the OTDR and the optical splitter can be eliminated by adjusting the whole curve up or down so that the peak for the reference signal reaches the same value in all subsequence OTDR readings. As shown in Figure 3, there are two additional peaks arising from the optical splitter. One is at the connection between the OTDR and the optical splitter, and the other is at the outlet of the optical splitter. Also, the two small peaks located between sensors 1 and 2, 4 and 5 are due to the connection between the extension optical fiber cord and the packaged sensor.

## 3. Field Trial of the Sensor

A field trial was arranged to test the performance of the sensor in aggressive environments. Ten sensors were installed in two piers of a footbridge located at the seafront of the southern part of Hong Kong Island. Under normal high tide conditions, the piers of the footbridge are partially submerged in seawater. Also, wind carries chloride ions and moisture to the surface facing the sea. The footbridge was constructed about thirty years ago and it has shown severe deterioration such as cracks, concrete spalling and steel rusting. It was repaired several years ago, but rust stains have re-appeared in some parts of the piers, which indicate the recurrence of steel corrosion. The field trial had three objectives: (i) to demonstrate the applicability of the sensors to existing structures; (ii) to assess the ability of the sensor to detect the corrosiveness of the environment in a practical situation and (iii) to prove the robustness and durability of the sensor, including its communication cord, under extreme environmental conditions.

### 3.1. Sensor Installation

**Figure 4.**
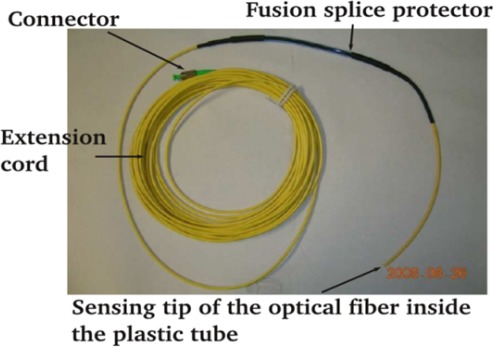
Packaged fiber optic corrosion sensor.

**Figure 5.**
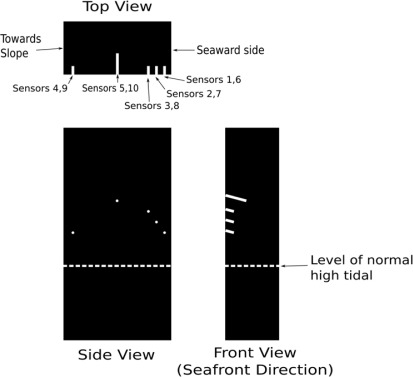
Locations of the sensors in the pier.

Ten sensors were installed in drilled holes on two adjacent piers. The details of the sensor packaging technique can be found in [10]. The sensor was packaged inside a 3 mm diameter rubber tube (Figure 4) which was then installed in a drilled hole of the pier with 10 mm diameter. The locations of the five holes of the piers are shown in Figure 5. As chloride penetration mainly occurs from the surface facing the sea, the holes were drilled from a perpendicular surface so the sensors are lying parallel to the seaward surface. The hole was sloping downwards slightly (about 15 to 20 degrees) to facilitate the penetration of grout for proper filling. All the sensors were installed above the level of normal high tide line. However, waves might be high enough to flood the sensors during storms and typhoons. The field trial would therefore test the sensors and the extension cords for their survival under extreme *in situ* conditions. After drilling, each hole was cleaned by compressed air and wetted by a film of water. The packaged sensor was then installed and grout was applied with a syringe. The grout was mixed cement *in situ* with a water-to-cement ratio of 0.7 and the addition of a viscosity modifier to prevent bleeding. Two hours after the hole was completely grouted, rapid-hardening epoxy was painted onto the surface of the grouted hole. After the hardening of epoxy, the surface of the hole was further protected by painting a layer of waterproof coating. With the sealing procedure, the grout (which is of high water-to-cement ratio) and its interface with the existing concrete would not act as preferential paths for the penetration of chloride ion and moisture. The sensors would then monitor the existing condition in the pier or the penetration of the corrosive agents from the seaward side if the surrounding environment of the sensors was not corrosive when they were installed.

Three sensors were installed at 60 mm (sensors 1 and 6), 80 mm (sensors 2 and 7) and 100 mm (sensors 3 and 8) from the seaward surface. The depth of each hole (taken as the horizontal distance from the bottom of the hole to the side surface) was 40 mm. According to the design drawing of the footbridge provided by the owner (which was a government office), the concrete cover to the longitudinal reinforcements was 50 mm on each side. In our original plan, the holes were drilled within the cover to a depth higher than 50 mm, so we would be detecting chloride penetration from the seaward surface as the sensor tip was closer to this surface than the side surface. However, there was a concern of the owner that the drilling of holes in the relatively thin cover might cause significant damage and surface spalling. As a result, the holes were drilled at the locations behind the steel reinforcement. Also, as the reinforcement density was rather high near the seaward surface, the drilling was stopped within the concrete cover, so we would not hit the steel reinforcements and introduce damages. As rust stains were observed at the seaward surface, the chloride concentration within the cover should be high enough to induce corrosion. We therefore expected to detect corrosion with sensors 1–3 and 6–8. As higher chloride concentration should exist for the sensor closer to the seaward surface, it was likely that corrosion would be detected in sensors 1 and 6 first. Sensors 4 and 9 were installed at the opposite side of the seaward surface. The depth was also 40 mm. At this location, the amount of chloride ion and moisture from sea spray should be lower than those in the seaward side. Corrosion therefore started later. According to the design drawing, the reinforcement density at the middle of the pier is very sparse. After verifying the actual rebar position by ferrodetector, sensors 5 and 10 were installed at a location 300 mm from the seaward side and at a depth of 100 mm. We expected that there should be very low chloride content so the sensors should not detect any corrosion. Hence, sensors 5 and 10 can test the *in situ* durability of sensors under extreme environmental conditions. All the above arguments related to the trend of the results will be assessed by the data collected by the *in situ* corrosion sensors.

### 3.2. Results and Discussions of the Field Trial

**Figure 6.**
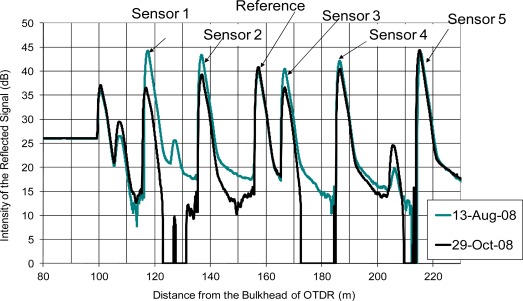
Typical comparison between two OTDR readings.

Figure 6 shows two OTDR readings. The sensors were installed on 13 August 2008. On 29 October 2008, which was 77 days after the installation, the OTDR reading showed a significant drop of the reflected signal from sensors 1, 2 and 3. Also, the peak of the reference, which measured the reflected signal of a cleaved optical fiber without coating also showed fluctuation. It was difficult to deduce whether the coating of the sensor was completely depleted directly from the OTDR reading. A better way to interpret the OTDR readings is to measure the change in the peak level of each sensor compared with the peak level after the installation. The change in the peak of the reference point indicated variation of insertion losses at each measurement. The OTDR reading should be adjusted up or down so that the peak levels of the reference point were identical for different OTDR readings. To account for the uneven distribution of the optical splitter among each channel and the variation of the quality of the coating, the baseline of each channel was first determined by measuring the peak level of a cleaved optical fiber without coating connected to the same extension cord at the channel. For a cleaved optical fiber without coating, the theoretical reflectivity with respect to air and water are −14.4 dB and −26.1 dB, respectively (Note: depending on the moisture condition inside the concrete, the fiber end should be in contact with either air or water.) The coating increased the reflectivity at the sensing point and it could be deduced by measuring the difference between the peaks of coated and uncoated optical fibers.

**Figure 7.**
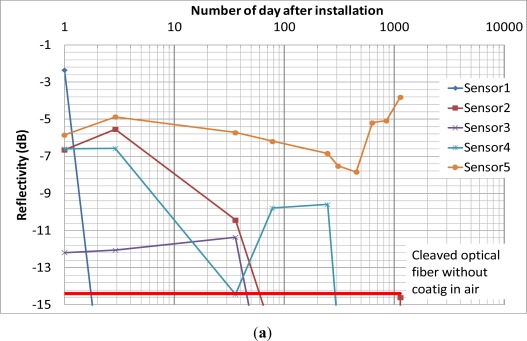

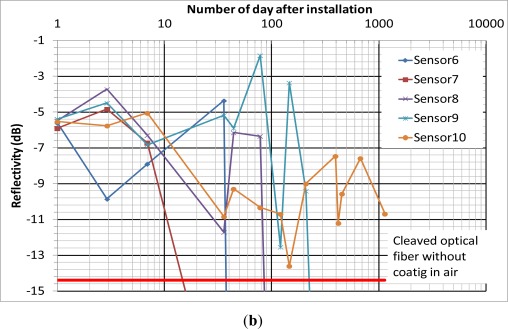
Results of the field trial test. (**a**) Sensors 1–5; (**b**) Sensors 6–10.

Figure 7 shows the results of the field trial over 1,189 days (more than 3 years). From Figure 7(a), the coating of sensor 1 was completely depleted in 2 days. The coating of sensors 2 and 3 were completely depleted between 35 and 78 days. The coating of sensor 4 showed significant drop at the 35th day but the subsequence reading showed that the coating was still intact and it was completely depleted after 247 days from installation. The reason might be significant insertion loss induced between the sensor and the extension cord during that reading. From Figure 7(b), the coating of sensors 6, 7 and 8 were completely depleted after 35, 6 and 77 days, respectively. The coating of sensor 9 was completely depleted after 208 days from installation. Although there was fluctuation of the reflectivity of sensor 5 and 10 during the monitoring period, the reflectivity showed that the iron coating of sensors had not been completely depleted after more than 3 years. It partially verified the durability of the sensor in a noncorrosive *in situ* environment.

**Figure 8.**
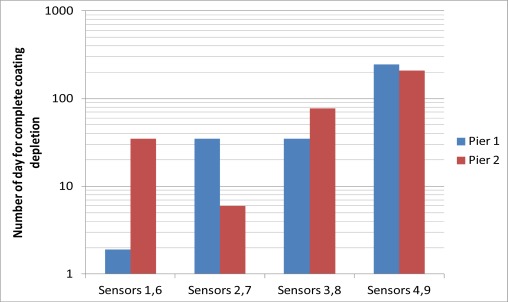
Summary of the number of days of the complete depletion of the coating in the field trial.

Figure 8 summarizes the number of days after installation for the last reading before the coating had been completely depleted. The trend generally agrees with the discussion in Section 3.1. The coating of sensors 1, 2, 3, 6, 7 and 8, which were installed near the seaward side surface, were completely depleted within 3 months, while sensors 4 and 9, which were installed at locations where the chloride concentration should be much lower than the seaward side, had their coatings completely depleted after 200 days. However, one can see variations in the trend for the two piers. Among sensors 1, 2 and 3, depletion of coating occurred earlier for a sensor closer to the sea. However, among sensors 6, 7 and 8, the coating was completely depleted for sensor 7 at an earlier time than sensor 6 (which is closer to the sea). In principle, the thin iron film should be depleted within a very short time if the chloride content is higher than certain threshold value. The rust stains at the seaward side surface indicated that the chloride content was already higher than the threshold value. The difference between the number of days required to completely deplete the coating was due to the variation of the distance between the sensor tip and the interface of the drilled hole. During sensor packaging, the sensor tip was embedded within 1 or 2 mm from the end of the protective rubber tube, but the actual distance might vary. Also, during the injection of the grout into the drilled hole, the packaged sensor might be pushed away from the interface. In addition, the packaged sensor might not be aligned perfectly perpendicular to the interface in a hole drilled at an angle. The actual time required to completely deplete the sensor coating depends on both the chloride content at the surrounding environment of the drilled hole and the travel distance to the sensor tip. From the results, it is likely that the fiber tips for sensors 1 and 1 and 7 are closer to the interface of the holes. Following the above arguments, the corrosion sensor does have a limitation in its accuracy and consistency. However, as corrosion is a very slow process, delaying the detection by even a few months is not a real concern. The important point is that one can clearly see the difference in results between sensors near the sea (sensors 1–3 and 6–8) and those on the opposite side (sensors 4 and 9) where the chloride content should be lower. Also, at the locations with little chloride content, where sensors 5 and 10 were placed, the sensors would not give false alarm due to their own deterioration under the concrete environment. Before closing, it should be mentioned that at least six strong tropical cyclones approached Hong Kong during the monitoring period, when business and schools were all closed and everybody stayed at home. The sensors are still able to provide signals under these conditions, showing the robustness of the packaging and installation techniques.

## 4. Accelerated Life Test of the Sensor

### 4.1. Objectives and Experimental Procedures

From the field trial, sensors 5 and 10 survived after more than three years from installation in a depassivating *in situ* environment. To an extent, this indicates the durability of the sensors. In practice, the steel corrosion of reinforced concrete structures under normal condition may take tens of years. That means the corrosion sensor may be in dormant state for a long period of time. Although the corrosion rate is very slow in highly alkaline environment, the thin iron film of the sensor (about 200 nm) may still be depleted before the actual corrosion of the reinforcement. Also, the silica of the optical fiber may be attacked in highly alkaline environment. Hence, it is essential to estimate the “life” of the sensor in the concrete environment. Since it is impractical to perform durability test over such very long periods, accelerated life test is performed. By elevating the temperature, the time required for chemical degradation process can be reduced at a rate that can be approximated by Arrhenius relationship [11]. That means the chemical kinetic in logarithmic scale is linearly proportional to the inverse of the absolute temperature.

Three sets of samples were prepared for the accelerated life test. Four samples in each set were tested in an environmental chamber at elevated temperature. One packaged sensor was embedded in the middle of a cubic cement paste with dimensions 40 mm × 40 mm × 40 mm. The total twelve sensors were all from the same batch of sputtering with 200 nm thick iron coating and the same packaging method. The water-to-cement ratio of the cement paste was 0.5. The cube was submerged in a water bath to maintain the humidity inside the hardened cement paste. Constant temperature was controlled for the first, second and third set of sample at 60, 70 and 80 degrees Celsius, respectively. The OTDR read the reflectivity of each sensor until the reflectivity returned to the level for no coating.

### 4.2. Results and Discussion

**Figure 9.**
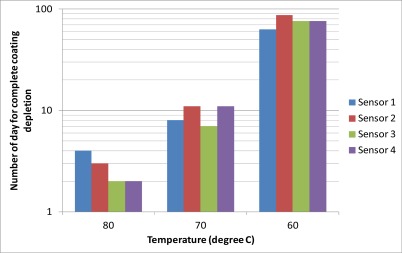
The results of accelerated life test.

**Figure 10.**
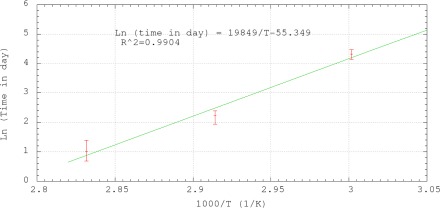
Arrhenius plot of the results of the accelerated life test.

Figure 9 shows the results of the accelerated life test of the sensors. The reflectivity of each sensor was monitored during the test as in Section 3.2. The number of day for complete depletion of the sensor coating was obtained from the last reading when the reflectivity was still higher than −14.4 dB. The averaged numbers of days for complete coating depletion for 60 °C, 70 °C and 80 °C were 3, 9 and 76, respectively. To extrapolate the results of the accelerated life tests to room temperature, the Arrhenius relationship was plotted in Figure 10. The horizontal axis of the plot is the reciprocal of the absolution temperature. The vertical axis is the natural logarithmic of the number of day required for complete coating depletion. By the linear best-fitted line as shown in Figure 10, the expected life of the sensor against chemical degradation at 10 °C, 20 °C and 30 °C are 7255, 626 and 63 years, respectively. The results show that the expected life against chemical degradation of the sensor in passivating environment under normal atmospheric temperature is at least comparable to the life of common structures, which is about fifty to seventy years.

## 5. Freeze-Thaw Cycling Test of the Sensor

### 5.1. Objectives and Experimental Procedures

**Figure 11.**
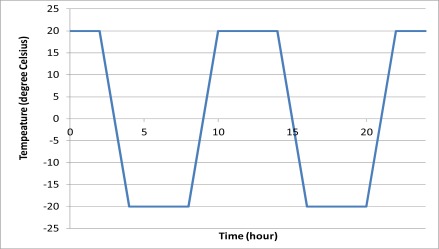
Daily temperature variation of the freezing thawing cycle.

Apart from the chemical degradation, the sensor may be subjected to physical degradation. One common physical degradation is freeze-thaw cycling. For hydraulic structures in cold areas, the frozen pore water expands and induces tensile stress near the pore during the low temperature period and it is released when the temperature rises above the freezing point of pore water. This does not only damage concrete, but also may damage the coating of the sensors. Four packaged sensors were embedded near the centroid of two identical mortar cubes with dimension 70 mm × 70 mm × 70 mm. The mixing ratio of the mortar water-to-cement-sand was 1:0.5:3. After normal curing, the mortar cubes were tested in a programmed environment chamber. The minimum and maximum temperatures of the freeze-thaw cycle were ±20 °C. The daily temperature variation in the environmental chamber is shown in Figure 11. The relative humidity above zero-degree Celsius was kept at 98%. The time for changing temperature level was two hours varying linearly with time while the temperature was held constant at the extremes for four hours. The whole freeze-thaw cycle took twelve hours. The reflectivity of each sensor was measured for about 350 freeze-thaw cycles.

### 5.2. Results and Discussion

**Figure 12.**
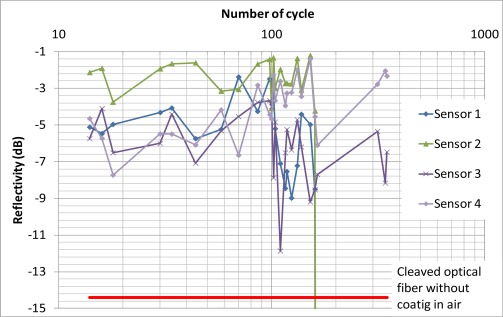
Results of freeze-thaw cycling test.

Figure 12 shows the reflectivity of each sensor over 348 freeze-thaw cycles. The red line indicates the reflectivity of a cleaved optical fiber without coating in air. The results showed that the coating of sensors 1 and 2 were damaged after the 160th cycle while the reflectivity of sensors 3 and 4 was still much higher than −14.4 dB after 348 freeze-thaw cycles. Both sensors 1 and 2 were in the same mortar cube. Although there was not any observable crack at the surface of the mortar cube, there might be some microcracks induced near the sensor and they damaged the sensor after 160 freeze-thaw cycles. In standard concrete testing, samples are considered durable against freeze/thaw if they can sustain more than 300 cycles. Two out of the four sensors are able to survive close to 350 cycles. With further improvement in the packaging technique, it may be possible to apply the sensor to cases where there is coupled freezing/thawing and chloride penetration to induce steel corrosion. This will be left as future work.

## 6. Conclusions

This paper presents the field trial, accelerated life test and freeze-thaw cycling test of a fiber optic based corrosion sensor. The method of multiplexing several sensors by OTDR and interpreting the OTDR readings of the multiplexed sensors is introduced. The installation procedures described in the field trial demonstrate the applicability of the sensors to existing structures. For the field trial results, the time required for complete depletion of the sensor coating is found to be related to the chloride content of the surrounding environment of the sensors. In a depassivated concrete environment, the sensors in the field trial survived at least 1,138 days (over 3 years). From the accelerated life test, the expected life against chemical degradation of the sensor at normal atmospheric temperature is estimated to be more than 60 years. From the freeze-thaw cycling test, the sensor can survive between 160 and 350 freeze-thaw cycles.
